# Recruited monocytes repair infections

**DOI:** 10.1002/ctm2.1121

**Published:** 2022-11-24

**Authors:** Rachel M. Kratofil, Paul Kubes

**Affiliations:** ^1^ Department of Physiology and Pharmacology University of Calgary Calgary Alberta Canada; ^2^ Calvin, Phoebe, and Joan Snyder Institute for Chronic Diseases University of Calgary Calgary Alberta Canada

## 1 MODELING STAPHYLOCOCCUS AUREUS INFECTIONS

*Staphylococcus aureus (S. aureus)* skin infection models have been widely used by researchers for decades in the fields of immunology and microbiology to study host‐pathogen interactions. However, despite thousands of published articles on *S. aureus* skin infections, we still do not fully understand how infections resolve in vivo. Furthermore, the consensus is that most researchers most often infect mice using high doses of bacteria in planktonic or free form (summarised in Ref. ^1^), at concentrations (millions to billions of CFU) that cause enormous tissue damage. The high‐dose infection causes a spectacular neutrophil response that is already well characterised.^2^ In this unrealistic infection scenario, neutrophils cause extensive bystander tissue damage while no or very few other myeloid cells including monocytes are recruited. The prevailing view is that the few monocytes somehow help neutrophils eradicate the infection. We decided to utilise a low inoculum of *S. aureus* (500 CFU) and attach it to an agar bead to model a foreign‐body infection. This infection model formed a biofilm which is common for *S. aureus* during clinical infections and surprisingly recruited equal numbers of both neutrophils and monocytes to the infection site. While neutrophils were responsible for capturing and killing *S. aureus*, the functional role for recruited monocytes was not known.

## 2 VISUALISING THE IMMUNE RESPONSE TO INFECTION AND TISSUE REPAIR

Our group had previously investigated the recruitment, fate and function of various immune cells during a sterile injury using a small thermal injury model in the liver.^3^, ^4^ Following liver injury, innate immune cells such as neutrophils and monocytes were recruited to the site of injury, and while neutrophils cleared debris from the injury site, monocytes surrounded the injury and were shown to convert from a classical to alternatively activated monocyte critical for tissue repair.^5^ Intravital microscopy of the bacterial infection in the skin revealed a revealed a differential localisation between neutrophils and monocytes: neutrophils inside and monocytes surrounding the infection site, and correspondingly only neutrophils interacting with *S. aureus*. By tracking the monocyte fate, we visualised the monocytes maturing to macrophages and identified these monocyte‐derived cells moved into the injured site well beyond the time‐course of bacterial clearance. In our study, we used complimentary methods of genetically modified mice, which lacked the chemokine receptor CCR2 involved in monocyte recruitment, as well as anti‐CCR2 depletion which depleted monocytes from circulation, to interrogate the functional role of recruited monocytes. In these monocyte‐deficient mice, bacterial clearance was equally efficient regardless of presence of absence of monocyte raising the question what are monocyte‐derived macrophages doing at the wound.

## 3 UNEXPECTED ROLE OF HUNGER HORMONES IN HEALING

Imaging these wounds at 14 days post‐infection revealed that the wounds of monocyte‐deficient mice were hypervascularised, and wounds were unable to heal at 30 days post‐infection indicative of a aberrant healing and a perpetual wound in monocyte‐deficient mice. The delayed healing phenotype at 30 days post‐infection with a thick collagen capsule was evident in the CCR2‐deficient mice. Furthermore, many of the CCR2‐deficient wounds were still hypervascularised at 30 days post‐infection. This was an interesting observation since patients with chronic, hypertrophic scars or keloid scars are often hypervascularised compared to normal skin.^6^

We pursued this mechanism of dysregulated angiogenesis and delayed healing and found that leptin, a hormone involved in satiety and metabolism, was over‐produced by adipocytes in the wounds of monocyte‐deficient mice. Leptin was able to directly induce angiogenesis in the wounds of wild‐type mice by acting on leptin receptor positive endothelial cells. The angiogenic response was able to be reduced in monocyte‐deficient mice by administrating a leptin antagonist, as well as the counteracting hormone, ghrelin. Unexpectedly, the recruited monocytes were a source of ghrelin at the site of infection and by performing bone marrow transfers of ghrelin‐deficient bone marrow into wild‐type recipients, we showed that immune‐derived ghrelin was critical for angiogenesis and healing.

## 4 FUTURE OUTLOOK

The results of this study may extend to cancer. Excessive angiogenesis is observed in solid tumours, where tumour progression from benign to malignant tumours can often be associated with an angiogenic switch from quiescent to proliferative vasculature.^7^ This angiogenic switch is often driven by VEGF but also other angiogenic mediators such as inflammatory cytokines, MMPs, and adipokines.^7^ In our protein screen for inflammatory mediators, MMPs and other angiogenic factors, we did not see any differences except for increased levels of leptin in wounds of CCR2‐deficient mice compared to wild‐type mice. Indeed, leptin‐induced angiogenesis is a common occurrence in tumours and leptin levels are elevated in several types of cancers from human patients.^8^

**FIGURE 1:**
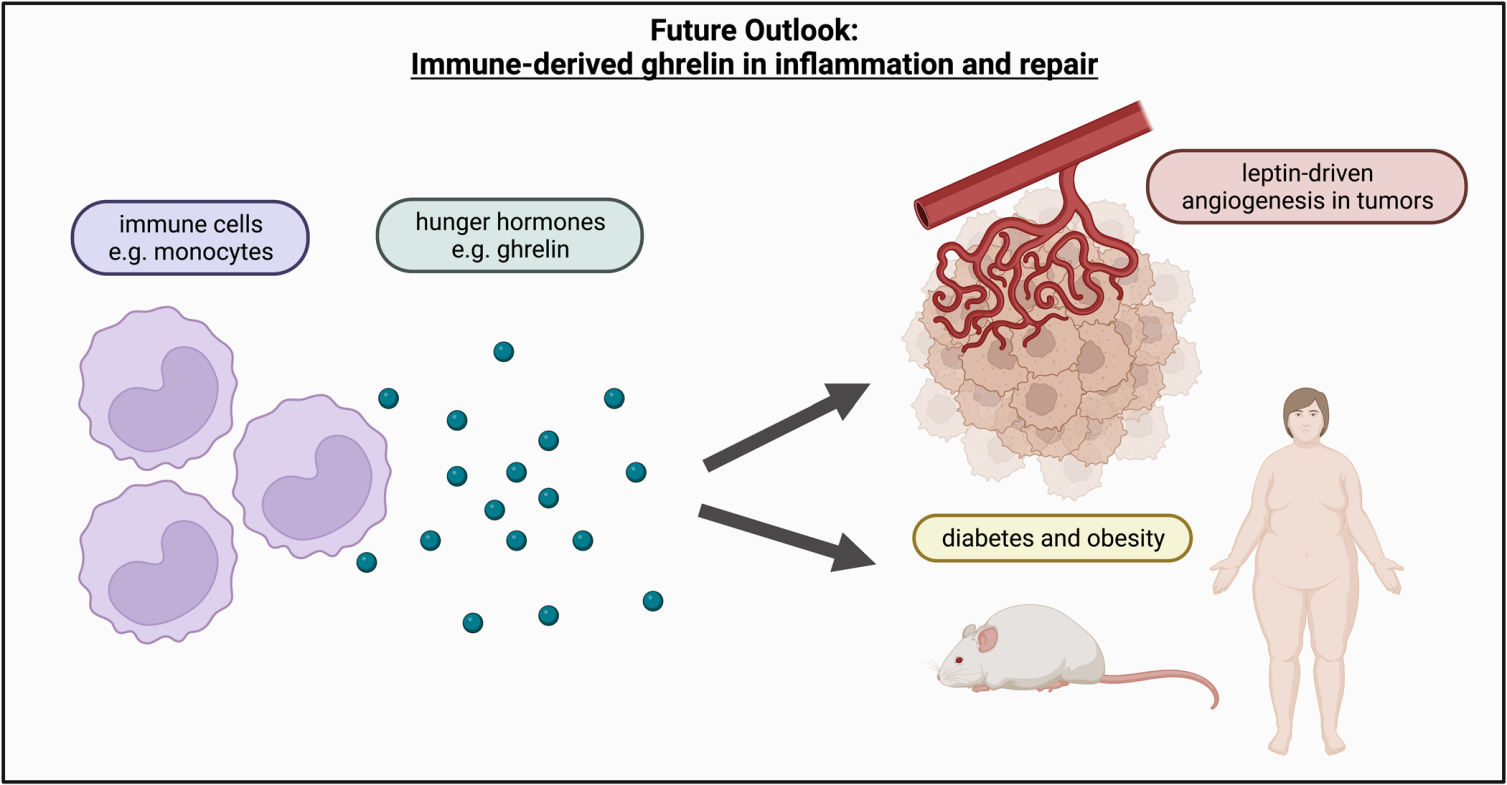
Immune‐derived ghrelin in inflammation and repair. Immune cells such as monocytes produce hormones such as the hunger hormone ghrelin which may have an effect on leptin‐driven pathologies such as angiogenesis in solid tumours as well as patients with metabolic diseases such as diabetes and obesity. Created with Biorender.com.

Our findings that monocyte‐derived ghrelin is critical for regulating angiogenesis in a mouse model of infection may be translatable to the human context considering human monocytes have been shown to express ghrelin at the mRNA level^9^ (Figure 1). However, more studies are needed to identify monocyte populations at sites of human *S. aureus* skin infections, and this can be done by collecting tissue biopsies of skin infections and performing immunostaining for monocytes and ghrelin. We would predict that patients with increased levels of leptin, for example obese patients or patients with metabolic disorders might be more likely to have less efficient wound healing. Indeed, it is well known that patients with chronic diseases and co‐morbidities are at a higher risk for *S. aureus* infections.^10^ For example, these patients are more likely to develop diabetic foot ulcers which often leads to a chronic wound that does not heal. Future directions using the *S. aureus* bead infection model would be to determine whether co‐morbidities such as diabetes or obesity would further delay bacterial clearance and/or tissue repair.

## CONFLICT OF INTEREST

The authors declare no conflict of interest.
